# A Retrospective Study Reviewing Timing to Onset of Habitual Psychogenic Non-Epileptic Seizures in a Home Video Telemetry Cohort

**DOI:** 10.3390/brainsci14121187

**Published:** 2024-11-26

**Authors:** Jade Cooper, Helen Chester, Arianna Fozzato, Elisaveta Sokolov

**Affiliations:** 1 Guy’s and St Thomas’ NHS Foundation Trust, London SE1 7EH, UK; jade.cooper@gstt.nhs.uk (J.C.); helen.chester@gstt.nhs.uk (H.C.); arianna.fozzato@gstt.nhs.uk (A.F.); 2 School of Optometry, Aston University, Birmingham B4 7UP, UK; 3 Cleveland Clinic, London SW1X 7HY, UK

**Keywords:** psychogenic non-epileptic seizures, functional neurological disorders, home video telemetry, video EEG, clinical neurophysiology

## Abstract

Objectives: This study aimed to investigate the onset time to habitual psychogenic non-epileptic seizures (PNES) in adults referred to Guy’s and St Thomas’ Neurophysiology Department for home video telemetry (HVT) with a clinical question of PNES. The primary objective was to determine the optimal time window for HVT recording for patients with suspected PNES to try to improve the allocation of clinical resources. The secondary objective was to explore any potential association between time to habitual PN ES onset and demographic indexes and other clinical, neuro-radiological and semiological findings. Methods: We performed a retrospective analysis of our XLTEK database between 2019 and 2020. A multifactorial analysis of PNES semiologic subtypes, patient demographics, psychiatric comorbidities and neuroimaging was conducted to explore their impact on time to PNES within an HVT study. People who had at least one typical PNES during their recording were included. The exclusion criteria included people who had the test performed without video recording. The total number of participants was 37. The data were extracted from our local XLTEK database. Statistical analyses using Mann–Whitney U and Fischer exact tests were carried out. Results: The mean time to first habitual PNES onset was seven hours, with a mean recording duration of 46 h. The most commonly occurring event type was blank spells (12, 32%), with the least common presentation being déjà vu (1, 3%). There was a significant association between time to PNES onset and male sex (*p* = 0.04). There was a significant association between time to PNES onset and abnormal MRI findings (*p* = 0.02). Particular PNES semiologic subtypes were not significantly linked with PNES onset time. Conclusions: Our study highlights that on average, patients with PNES will rapidly have their first habitual event within an HVT study (mean time to event onset of seven hours), consistent with the current literature. This raises the question of whether HVT study duration could be reduced to release study resources and aid departmental efficiencies. We also observe the novel finding that men presented significantly earlier with their habitual PNES event than women, and that abnormal imaging findings were also significantly associated with an earlier time to event onset, although the reason for this association is yet to be determined.

## 1. Introduction

Psychogenic non-epileptic seizures (PNES) are the most common presentation of functional neurological disorders (FNDs) [1]. PNES is characterised by paroxysmal events, which can be associated with motor, sensory and/or autonomic manifestations, as well as other cognitive manifestations. These clinical manifestations occur in the absence of electroencephalographic (EEG) changes associated with epileptogenic activity [1,2].

PNES is a relatively common disorder, with an estimated prevalence of 10.6/100,000 for the highest level of diagnostic certainty (diagnosed using video EEG [3]) according to a recent cross-sectional study, and an annual incidence of 1.4–4.9 cases/100,000 people/year [4]. PNES is an imitator of epileptic seizures in one-third of cases of patients with dual diagnosis [5] and is reportedly more common in females [6]. There is a scarcity of articles describing PNES semiology, and an estimated 20–30% of epilepsy cases are misdiagnosed PNES [7]. 

Interestingly, a range of predisposing, precipitating and perpetuating factors has been identified in the aetiology of PNES [8,9,10]. In particular, an association with psychiatric comorbidities, including mood, anxiety disorders and borderline and depressive personality disorders [11], has been noted. Moreover, other medical comorbidities, including epilepsy [5], structural brain abnormalities [5], a history of brain surgery [5], febrile convulsion, status epilepticus and the use of combined (≥3) antiepileptic drugs (AEDs), have also been proposed as potential risk factors for PNES in patients with epilepsy [12]. An association between PNES and traumatic brain injury is also noted [1,13].

There is a clinically urgent need to improve early and accurate PNES diagnosis; this is critical to offer access to the correct treatment options and reduce potential harm to patients. It is important to differentiate epileptic from non-epileptic events. Misdiagnosed PNES could lead to unnecessary AED administration, diagnostic testing and sometimes even emergency medical procedures for the management of status epilepticus (SE). In PNES patients, these interventions can increase morbidity and even mortality [14,15,16]. A recent study has revealed that PNES patients have a standardised mortality ratio 2.5 times higher than that of the general population; this is a similar rate to patients diagnosed with drug-resistant epilepsy [14,15,16]. Despite the existence of clear guidance of standards for PNES diagnosis, there is an average diagnostic delay of 7.2 years [17]. Moreover, standard recording protocols and guidelines for video electroencephalography (vEEG) in suspected PNES, including ideal recording duration, are yet to be determined. This renders the diagnostic procedure for PNES more challenging and can also lead to the poor optimisation of hospital equipment, including home video telemetry (HVT) equipment, and potentially a poorer diagnostic experience for the patient [18,19]. HVT is one of the primary diagnostic modalities used in PNES. 

For these reasons, improving access to timely diagnosis for PNES is paramount, and to achieve this, it is vital to improve the allocation of limited clinical resources available to neurophysiology departments. The primary aim of this study was to establish the time to habitual PNES onset to optimise the required HVT duration and improve accessibility to HVT testing for suspected PNES to ultimately expedite diagnosis and improve health outcomes. At present, there is no recommendation for vEEG recording time according to the referral question. The authors hypothesised that patients presenting with PNES would have their typical event in less than 24 h.

In addition to this, the secondary aim of this study was to categorise PNES type based on the semiology of events captured during the HVT recording and explore whether there was any relationship between PNES type and time to event onset. Our current understanding of the semiological characteristics of PNES is limited and is complicated by a significant overlap with epileptic seizures and mixed seizure disorders. Whilst some semiologic features can be useful to differentiate PNES from epileptic seizures, PNES is not always stereotyped across patients. Two commonly utilised semiologic classification systems for PNES [20,21] demonstrate high variability when assessing the semiology of PNES [21,22]. We consider the semiologic subsets of PNES in this patient cohort and investigate their relation to the timing of event onset. 

## 2. Materials and Methods

**Study Aims:** This retrospective study reviews a 50-patient cohort who underwent HVT at Guy’s and St Thomas’ NHS Foundation Trust in the Department of Clinical Neurophysiology between November 2019 and November 2020. The primary aim of this study was to quantify the mean duration of the time to onset of the first habitual PNES in this cohort. The secondary aim was to identify and categorise event types and consider their association with mean time to event onset. 

**Patients:** A sample of 50 patients who were referred with the question of PNES during their HVT recording were identified between November 2019 and November 2020. This patient number was attained in order to adequately power this study.

**Exclusion Criteria:** Patients who did not have video recording, or who had inadequate video recording (patient not clearly visible for most of the recording), were excluded from the final analysis. This was due to a requirement to analyse event semiology, which cannot be achieved without a video recording (N = 6/50). Patients who did not have a typical PNES during the HVT were excluded from the final analysis (N = 7/50). A total of thirteen patients were excluded from the final sample (N = 37).

Equipment and Setup: The HVT studies were carried out using XLTEK/Natus, as per departmental protocol. Ambu silver/silver chloride disposable electrodes were attached using the 10–20 International System. Elefix conductive paste was used to secure the electrodes, with 3 cm Hypafix tape placed over the electrode to secure. A double 6-size Surgifix netting with a retaining bandage tied underneath the chin was used to prevent electrode movement. Bilateral mastoid (A1 & A2) ECG and bilateral deltoid EMG electrodes were utilised for all patients. Electrodes were then secured in a shoulder bag for comfort, and the patient took the equipment (camera, batteries and infrared light) home for video monitoring. The patient was supplied with verbal, visual (pictures) and written instructions on how to set up the video camera and infrared light at home. This involved ensuring the camera was plugged into a mains electrical source to ensure battery life was maintained, switching the camera on by opening the LCD video screen and pressing the red record button. At nighttime, the patient was instructed to switch the video camera to ‘night mode’ and use the supplied infrared light. The infrared light was set up by inserting two AA batteries and switching it on. This did not illuminate the room.

Following the return of the HVT kit via courier to the hospital, all data were uploaded and reviewed. A factual report was written by a physiologist and a conclusion was written by the Neurophysiology Consultant. 

**Data Collection:** The data were retrospectively collected from the written HVT reports. The data collected are outlined in Table 1. The event description was extracted from the report and subsequently categorised. Additional data regarding patient demographics and relevant medical history were acquired from the Electronic Patient Record (EPR) System. The EEG data were extracted from the local XLTEK database on site at St. Thomas’ Hospital, exported and later numerically coded into a Microsoft Excel spreadsheet for analysis. All data were anonymised prior to data analysis.

**Statistical Analysis:** Data were collected from the described group of patients who all underwent an HVT study. Of particular interest were the occurrence and characteristics of a PNES attack. 

**Statistical Methods:** A first set of analyses examined associations between patient factors (as per Table 1, Table 2, Table 3, Table 4, Table 5, Table 6 and Table 7) and the time to first PNES. Individual sample size is denoted (as per Table 1, Table 2, Table 3, Table 4, Table 5, Table 6 and Table 7). All patient factors were categorical in nature, and the difference in the time to PNES between groups was examined using the Mann–Whitney U test. The association between a psychiatric comorbidity and time to first PNES was further examined by adjusting for two pre-defined variables (psychiatric comorbidity and AED use). This analysis was performed using linear regression. Due to the skewed distribution of the time to PNES, this variable was given a log transformation before analysis. 

Additional analyses examined whether the occurrence of each PNES type varied between older (40 years or more) and younger patients (less than 40 years), and also between males and females. Due to the categorical nature of these variables, the analyses were performed using Fisher’s exact test.

## 3. Results

Data were collected from a total of 50 patients. Of these, 13 were excluded from the analysis, leaving 37 patients in the final analysis. Summaries of the characteristics of the patients in the analyses were made. Categorical variables are summarised by the number and percentage of patients in each category. Continuous variables were summarised by the mean and standard deviation if found to be approximately normally distributed, and by the median and interquartile range if not. 

The data suggested that the majority of the study participants were female (84%), with a mean age of 38 at the time of the HVT studies (Table 1). The most common seizure description based on the patient’s history was focal with an impairment of awareness, which accounted for three-quarters of patients. Only just over a third (38%) had an existing epilepsy diagnosis, and fewer (28%) had a family history of epilepsy. Over half (57%) were on AEDs (Table 1). We analysed the characteristics of the HVT studies, which are summarised in Table 2. The results suggested a mean duration of HVT of 46 h. A quarter of the patients experienced technical difficulties, of which video difficulties were the most common. The majority (over 90%) of patients were alert and cooperative before the study (Table 2).

All included patients underwent a typical PNES attack during the study. The median number of attacks was three, with these more commonly reported by the patient than by a witness. The majority (86%) were diurnal attacks. The median time to first habitual PNES was seven hours. There was a range of PNES types, with the most common being blank spells/vacant (32% of patients) and other reasons (37%) (Table 2).

Additional analyses examined associations with the type of PNES event experienced. As some patients had more than one type of event, separate analyses were performed for each type of event. As only one patient underwent a déjà vu event, no further analyses were performed for this event type.

Firstly, the time to first habitual PNES was examined for those with and without each type of event. The results are summarised in Table 3. The first figures are the number of patients with and without each type of event. The median and interquartile range time for each group are also presented, along with *p*-values indicating the significance of the differences. The results indicated that there was no strong evidence that any of the event types were associated with the time to first PNES (Table 3). There was slight evidence of an association for dizziness, although this result did not quite reach statistical significance. The trend was towards a shorter time for those with dizziness events, although it is noted that there were only two of these patients (Table 3). 

Analyses were also performed to compare the number of events between patients with and without different habitual PNES event types (Table 4). There was no evidence that the number of habitual PNES events varied significantly between patients with and without each type of event.

Next, we examined the association between a psychiatric comorbidity and each type of PNES event (Table 5). These Figures show the number of subjects with each type of PNES event for those with and without a comorbidity. *p*-values indicating the significance of the results are shown in the final column. 

The results indicated that a psychiatric comorbidity was not found to be significantly associated with any particular type of PNES event. We then examined the association between a number of patient factors and the time to first habitual PNES (Table 6). The number of patient factors for each subgroup was reported and analysed independently. The median and interquartile range time for each group are also presented, along with *p*-values indicating the significance of the differences. The results indicated that there was no significant association between AEDs, psychiatric comorbidity, age or attitude to HVT and the time to first habitual PNES. However, both gender and MRI findings were found to be significantly associated with the time to habitual PNES (Table 6). Males had a shorter time to first habitual PNES than females. The median time for males was five hours, which contrasted with a median time of eight hours for females. Patients with positive MRI findings also had shorter times. The median time to PNES was 4 h for those with abnormal MRI findings, compared to a median time of 10 h for those with normal MRI findings (Table 6). 

For the association between a psychiatric comorbidity, we considered how time to first habitual PNES varied between those with and without a comorbidity after adjusting for AEDs and use of psychiatric drugs. The difference between groups was not significant after adjusting for these variables (*p* = 0.82).

An additional analysis examined the difference in the number of habitual PNES between patients on AEDs and those not on AEDs. For the patients not on AEDs, the median number of PNES was 3 (IQR: 2 to 4.5), and for patients on AEDs, the median number of PNES was 2 (IQR: 2 to 4). This difference was not statistically significant (*p* = 0.54). Further, we examined the association between patient age and each occurrence of each type of habitual PNES event. The results indicated that age was not significantly associated with any type of habitual PNES event (Table 7). 

A similar set of analyses was performed to examine the association between gender and each type of habitual PNES event (Table 8). 

The analyses suggested no significant differences between males and females in terms of the types of PNES experienced. 

## 4. Discussion

The diagnosis of PNES remains challenging. Event semiology, psychiatric history, prolactin assay, seizure provocation techniques and psychological testing constitute the bulk of the information we use to support PNES diagnosis. However, there is a general agreement that video–EEG monitoring is the gold standard test in the diagnosis of PNES [23,24]. This retrospective study aimed primarily to quantify the time to habitual PNES onset in patients where this diagnosis was in question and who underwent HVT between November 2019 and November 2020 at our institution. The secondary aim was to categorise PNES types within this cohort and explore potential relationships between event type and patient-independent epidemiological factors. We hypothesised that the study duration could be shortened in this patient group based on whether the referral question was for PNES alone. 

An important distinguishing feature of PNES is that the duration is typically longer than ES, with episodes lasting >2 min on average, and with events lasting over 10 min that are not suspected status epilepticus being highly suspicious for PNES [23]. The time to event onset in PNES patients has not been explored in depth. Woollacott and collaborators [25] found in a retrospective analysis of 159 PNES patients that 25 had an attack prior to or during electrode application and the remaining 134 patients had an attack within 48 h. However, this study did not identify exactly when the events occurred within the 48 h. In addition, Perrin and colleagues [26] found that the median latency to first PNES in a retrospective study of 216 patients was 7 h. This study found that the time to first PNES was dependent upon the type, with later onset times in both the minor motor and subjective/experiential groups when compared with the major motor and akinetic groups, which occurred earlier [26]. Furthermore, a retrospective study found that the mean time to first event was 20.88 h for the PNES group (n = 111) and 30.99 h for the epileptic seizure group (n = 121). More than half (52%) of the patients with PNES, and about one third (38%) of the patients with epileptic seizures, had an event within the first 24 h [27]. Finally, Herskovitz found that duration of PNES was not consistent when patient’s had multiple events during a single video telemetry recording, however did not explore the time to event onset [28]. 

Our study demonstrated that the mean time to first habitual PNES onset was 7 h, with a mean total recording duration of 46 h. There are currently no established national guidelines in the United Kingdom (UK) regarding HVT EEG duration [29]. An average duration of 24–72 h across different UK providers is described [30,31,32]. Our finding has potential implications for amendments to future study durations based on the referral question. Considering our results, it seems reasonable that if the patient is referred with a strong likelihood of PNES, without a history of concomitant epileptic seizures, a study duration of 24 h is highly likely to be sufficient to capture and classify habitual PNES events. We suggest 24 h rather than a shorter duration in order for adequate sleep to also be recorded. The shorter study duration in this group would firstly be less onerous for the patient, with a reduced likelihood of technical issues, and additionally would ultimately release both equipment and staff sooner to carry out more studies; thus, overall, this change has the potential to streamline an HVT service and improve efficiency and quality of care for the patient. 

We also consider semiologic subtypes within our cohort. A semiological classification of PNES has the potential to aid the diagnosis and management of these patients, and also provide information to aid the standardisation of future diagnostic study protocols. Although not described in the literature as explicit categories, the existing body of evidence describes several unique PNES characteristics. These may include eye-opening behaviours [33] such as eye fluttering, geotropic eye movements and closed eyelids resisting passive opening [2,34]; additionally, motor signs are seen, often fluctuating in nature [35], including convulsive or thrashing episodes characterised by disorganised movements across all four limbs, side-to-side head movements, clenched jaw during tonic spells, pelvic thrusting and arched back. Moreover, “catatonic” episodes, marked by pseudo syncope, eye closure and unresponsiveness, are also seen in PNES [36,37,38]. Additionally, whole-body shaking or tremulous movements can be observed, which, in contrast to generalised tonic–clonic seizures (GTCS), do not involve rapid muscle contraction followed by relaxation. Asynchronous limb movements are also indicative of PNES [38], and a fluctuating course has been shown to be a strong, sensitive predictor for PNES [35]. 

Event-related moaning and weeping may also be seen [39] in PNES, and speech, if present, can be intelligible and feature partial responses to questions [5]. This differs from epileptic seizures, which are generally characterised by meaningless and monotone repetitions of phrases and sounds and an inability to engage [40]. Despite differing motor features, distinguishing PNES from focal seizures or absence seizures can be complex. Frontal lobe seizures and PNES may share elements of retained awareness and responsiveness, with associated hypermotor features [5]. 

Given the complexity and variability of the event semiology of our study cohort, we based our semiologic classification on a pre-existing PNES classification system that has subdivided PNES events into three main categories, namely motor seizures, non-motor seizures and mixed semiology [41]. According to this, in our cohort, we identified five sub-categories, which include blank spells/vacant (32%), convulsive (13%), jerks (27%), dizziness (5%) and other (27%) [42]. PNES subtypes vary between patients, and thus direct comparison is challenging [24,33,34,35,36,37,38,39,41,43,44,45,46,47,48,49]. The most commonly occurring PNES subtype according to our categorization was blank spells, with jerks being the second most common. Semi-rhythmic tremors have been reported more typically as the most common habitual PNES subtype, with only 11.2% of PNES patients in the same study presenting with habitual blank spells [20]. Head shaking and hip thrusting, as well as a fluctuating course, are semiological features commonly reported in PNES [35,36]. The terminology used to describe PNES subtypes varies, and thus direct comparison to prior studies is challenging [21].

PNES is three times more prevalent in female patients than in males [3,4], and the present cohort is reflective of this, with 87% of the studied cohort being female. We identified a statistically significant and novel association between time to first habitual PNES onset and gender, with male patients having the first habitual PNES earlier in the recording than female patients. The time of onset with respect to gender is not well understood and needs future consideration. The semiology differences between men and women have been explored, with men being shown to have clearer motor manifestations [48] and a shorter event duration [49]. Future research may wish to expand on such findings, in particular exploring whether there is a difference in suggestibility to PNES between male and female patients which could affect the time to habitual PNES onset.

It may also be pertinent to identify whether there is a difference between male and female comorbidities and PNES onset. In this cohort, 3/6 (50%) male patients had a psychiatric comorbidity, which included bipolar disorder, depression and ADHD, vs. 15/31 (48%) female patients. There did not appear to be a clear difference between these groups in the likelihood of a psychiatric comorbidity.

Pre-existing cortical structural abnormalities are a known risk factor for the development of PNES in patients with epilepsy [5,50]. A systematic meta-analysis demonstrated that there is a clear association between structural and functional brain abnormalities and PNES, although the brain regions involved varied between patients [50]. In our study, we identified several MRI changes, including hippocampal sclerosis, emarginated lesions, encephalomalacia and meningiomas. Our findings did not highlight a common brain area that might be associated with PNES. We conclude, in line with the current literature, that a patient has an increased incidence of PNES when they have any brain lesion [5,51]. The most common MRI changes occurred in the temporal lobes, noted in 44% of patients. The existing literature also concurs that the most commonly found MRI change in patients with PNES, if they have any MRI change, is in the temporal lobes [51]. Moreover, we demonstrated a significant association between an abnormal MRI finding and the time to PNES onset. Patients who had abnormal MRI brain scan findings were more likely to have their events earlier in the HVT recording than those who had normal MRI brain scans. This was not documented in the existing literature that we explored and is a novel finding of the present study. Future consideration as to the underlying pathophysiology linking structural lesions to an earlier time to PNES onset is needed. 

### 4.1. Study Strengths & Limitations

This study aligns with the previous literature in the field, indicating that patients with PNES are likely to have a habitual PNES in <24 h [25,26,27], and provides an up-to-date reflection of semiology and time to event onset in this PNES cohort. Equally, the cohort reflects the variability of PNES semiology, again in keeping with our understanding of PNES [23,35,37]. Therefore, our results provide additional supportive evidence with this reflective cohort for a reduced standard recording time in patients, as referred with the question of PNES. 

All of the patients in this study were self-reporting, and none of them had any supporting witness reports. This means that our data rely on the accurate reporting of events by the patient without the validation of a witness. The benefit of outpatient routine and sleep EEG recording when events are captured is that the physiologist is present to confirm the habitual nature of the event and perform an ‘ictal’ assessment. This is a current limitation of evaluating PNES semiology using HVT, in particular for the patients presenting with ‘blank spells’, which accounted for 32.4% of our study cohort. Ictal assessment in the HVT setting can be challenging, although with the advent of artificial intelligence tools, in due time we may be able to carry out real-time remote ictal assessments [52]. 

Thirteen patients within our initial sample were excluded due to video or camera problems. HVT can be limited by such factors. This provides an opportunity for local audit and protocol developments to explore how these issues might be prevented in the future.

Of note, the present study did not explore whether the MRI changes were present prior to or post-onset of PNES presentation. Future research may wish to determine whether the onset of PNES was pre- or post-MRI changes. 

### 4.2. Study Implications

Our study provides a comprehensive overview of the relationship between patients with suspected PNES and time to habitual event onset, identifying that on average, patients had their habitual PNES onsetting within seven hours of HVT recording. Such findings hold implications for revising local protocols for HVT duration based on the clinical question of PNES. This would support the preservation of resources and also allow additional testing to be performed, with resources potentially becoming available earlier. It is likely that this shorter study duration would also improve the patient experience; however, this needs to be considered objectively in future work. 

### 4.3. Conclusions

This retrospective analysis demonstrates that habitual PNES is captured within 24 h in adult patients undergoing HVT. On average, PNES events were captured within seven hours of starting the recording. This finding could help tailor HVT study durations in the future with the goal of making services more efficient and cost-effective, and could have the potential to improve the patient experience. The significant association between abnormal MRI brain findings and the time to PNES warrants further investigation, in particular focusing on the potential psychological manifestations a patient may experience when they are diagnosed with a brain lesion. 

### 4.4. Future Directions

In addition, future studies may wish to explore whether activation procedures were performed during the HVT setup and whether this may have an association with time to event onset. It would be interesting to identify whether suggestibility may impact the time to onset in PNES patients undergoing HVT.

Finally, prospective data collection may help to categorise PNES more accurately, with the opportunity to call patients or relatives for clarification of their events should this be required. Subjective event description by patients provides only a single view in PNES research [45,46], whereas a multi-dimensional approach may provide more clarity. The systematic classification of PNES semiology would aid research in this patient population; however, this is no easy feat with such variability in patient descriptions and how each individual feels they experience their events.

## Figures and Tables

**Table 1:** Demographic of participants in analysis. Summary statistics are mean ± standard deviation or number (percentage).

Variable	n (Sample Size)	Category	Summary (%)
Gender	37	Female	31 (84)
		Male	6 (16)
Age at onset	10		17.9 ± 13.1
Age at HVT	37		38.8 ± 14.4
Seizure type	37	GTCS	5 (14)
		TA	1 (3)
		MS	2 (5)
		Focal with LoA	28 (76)
		Multiple types	1 (3)
Seizure frequency	9	Daily	1 (11)
		Weekly	2 (22)
		Monthly	1 (11)
		Other	5 (56)
Existing epilepsy diagnosis	37	No	23 (72)
		Yes	9 (28)
Family history of epilepsy	32	No	23 (72)
		Yes	9 (28)
On antiepileptics	37	No	16 (43)
		Yes	21 (57)
Psychiatric comorbidity	37	No	19 (51)
		Yes	18 (49)
Psychiatric drugs	37	No	26 (70)
		Yes	11 (30)
Neuropsychology input	37	No	25 (68)
		Yes	12 (32)

**Table 2:** Home video telemetry studies. Summary statistics are mean ± standard deviation, median [interquartile range] or number (percentage). (*) Patients could indicate more than one technical difficulty (**) Patients could indicate more than one type of PNES. Percentage values may not add up to 100%.

Variable	n (Sample Size)	Category	Summary
HVT duration (h)	37	−	46 ± 14
Technical difficulties	36	No	27 (75%)
		Yes	9 (25%)
Specific difficulties (*)	9	Battery	2
		Video	7
		Electrode problems	2
		Removal before end	2
Attitude prior to study	31	Alert and cooperative	29 (94%)
		Anxious	1 (3%)
		Tearful	1 (3%)
PNES during HVT	37	No	0 (0%)
		Yes	37 (100%)
Number of PNES attacks	37	Patient reported	3 [2, 4]
	37	Witness reported	0 [0, 2]
	37	Total	3 [2, 4]
Timing of PNES	37	Diurnal	32 (86%)
		Nocturnal	5 (14%)
Time first PNES (h)	37	−	7 [4, 15]
PNES event habitual	37	No	2 (5%)
		Yes	35 (95%)
PNES type (**)	37	Blank spell/vacant	12 (32%)
		Convulsive	5 (14%)
		Jerks	10 (27%)
		Dizziness	2 (5%)
		Déjà vu	1 (3%)
		Other	10 (37%)

**Table 3:** Associations with time to first habitual PNES and PNES type. Time to first habitual PNES is reported as median and interquartile range (IQR).

Habitual PNES Type	Presence	n (Sample Size)	Time to First PNES (h) Median [IQR]	*p*-Value
Blank spell/vacant	Absent	25	7 [4, 18]	0.85
	Present	12	7 [5, 10]	
Convulsive	Absent	32	7 [4, 14]	0.69
	Present	5	9 [6, 13]	
Jerks	Absent	27	7 [5, 12]	0.81
	Present	10	9 [4, 19]	
Dizziness	Absent	35	7 [5, 16]	0.06
	Present	2	2 [0, 4]	
Other	Absent	27	7 [5, 12]	0.69
	Present	10	9 [4, 18]	

**Table 4:** Associations between number of habitual PNES events and habitual PNES type. Number of PNES events is reported as median and interquartile range (IQR).

PNES Type	Presence	n (Sample Size)	Number of PNES Events Median [IQR]	*p*-Value
Blank spell/vacant	Absent	25	3 [2, 4]	0.14
	Present	12	2 [2, 3]	
Convulsive	Absent	32	3 [2, 4]	0.63
	Present	5	2 [2, 3]	
Jerks	Absent	27	3 [2, 4]	0.86
	Present	10	3 [1, 12]	
Dizziness	Absent	35	3 [2, 4]	0.12
	Present	2	5 [4, 6]	
Other	Absent	27	2 [2, 4]	0.35
	Present	10	4 [2, 5]	

**Table 5:** Associations between psychiatric comorbidity and PNES type. Presence/absence of PNES type in relation to comorbidities is expressed as number of patients and percentage values.

PNES Type	Presence	No Comorbidity	Comorbidity	*p*-Value
Blank spell/vacant	Absent	14 (74%)	11 (61%)	0.50
	Present	5 (26%)	7 (39%)	
Convulsive	Absent	17 (89%)	15 (83%)	0.66
	Present	2 (11%)	3 (17%)	
Jerks	Absent	14 (74%)	13 (72%)	1.00
	Present	5 (26%)	5 (28%)	
Dizziness	Absent	17 (89%)	18 (100%)	0.49
	Present	2 (11%)	0 (0%)	
Other	Absent	14 (74%)	13 (72%)	1.00
	Present	5 (26%)	5 (28%)	

**Table 6:** Associations with time to first habitual PNES. Time to first habitual PNES is reported as median and interquartile range (IQR). (*) Analysis for patients undergoing an MRI only. Bold text indicates a statistically significant finding.

Variable	Category	n	Time First PNES (h) Median [IQR]	*p*-Value
On AEDs	No	16	8 [5, 15]	0.40
	Yes	21	6 [4, 12]	
Psychiatric	No	19	7 [4, 13]	0.92
comorbidity	Yes	18	7 [5, 16]	
Age category	≤40	22	8 [5, 12]	0.94
	>40	15	6 [4, 19]	
Gender	Female	31	8 [5, 18]	**0.04**
	Male	6	5 [3, 6]	
MRI findings (*)	Normal	13	10 [6, 12]	**0.02**
	Abnormal	7	4 [0, 7]	
Attitude to HVT	Alert	29	7 [5, 16]	0.81
	Anx./tearful	2	9 [7, 10]	

**Table 7:** Associations between age and PNES type are expressed as number of patients and percentage values.

PNES Type	Presence	Age ≤ 40	Age > 40	*p*-Value
Blank spell/vacant	Absent	13 (59%)	12 (80%)	0.29
	Present	9 (41%)	3 (20%)	
Convulsive	Absent	19 (86%)	13 (87%)	1.00
	Present	3 (14%)	2 (13%)	
Jerks	Absent	18 (82%)	9 (60%)	0.26
	Present	4 (18%)	6 (40%)	
Dizziness	Absent	20 (91%)	15 (100%)	0.51
	Present	2 (9%)	0 (0%)	
Other	Absent	18 (82%)	9 (60%)	0.26
	Present	4 (18%)	6 (40%)	

**Table 8:** Associations between gender and PNES type.

PNES Type	Presence	Female	Male	*p*-Value
Blank spell/vacant	Absent	22 (71%)	3 (50%)	0.37
	Present	9 (29%)	3 (50%)	
Convulsive	Absent	26 (84%)	6 (100%)	0.57
	Present	5 (16%)	0 (0%)	
Jerks	Absent	24 (77%)	3 (50%)	0.31
	Present	7 (23%)	3 (50%)	
Dizziness	Absent	30 (97%)	5 (83%)	0.30
	Present	1 (3%)	1 (17%)	
Other	Absent	22 (71%)	5 (83%)	1.00
	Present	9 (29%)	1 (17%)	

## Data Availability

The data presented in this study are available on request from the corresponding author.
