# Correction: Cooper et al. A Retrospective Study Reviewing Timing to Onset of Habitual Psychogenic Non-Epileptic Seizures in a Home Video Telemetry Cohort. *Brain Sci.* 2024, *14*, 1187

**DOI:** 10.3390/brainsci15010064

**Published:** 2025-01-12

**Authors:** Jade Cooper, Helen Chester, Arianna Fozzato, Elisaveta Sokolov

**Affiliations:** 1 Guy’s and St Thomas’ NHS Foundation Trust, London SE1 7EH, UK; jade.cooper@gstt.nhs.uk (J.C.); helen.chester@gstt.nhs.uk (H.C.); arianna.fozzato@gstt.nhs.uk (A.F.); 2 School of Optometry, Aston University, Birmingham B4 7UP, UK; 3 Cleveland Clinic, London SW1X 7HY, UK

## Missing Citation

In the original publication [1], reference number 12 is “Au Yong, H.M.; Minato, E.; Paul, E.; Seneviratne, U. Can Seizure-Related Heart Rate Differentiate Epileptic from Psychogenic Nonepileptic Seizures? *Epilepsy Behav.* **2020**, *112*, 107353”. This is incorrect and the correct citation linked to 12 should be “Yon, M.I.; Azman, F.; Tezer, F.I.; Saygi, S. The Coexistence of Psychogenic Nonepileptic and Epileptic Seizures in the Same Patient Is More Frequent than Expected: Is There Any Clinical Feature for Defining These Patients? *Epilepsy Behav.* **2020**, *105*, 106940. https://doi.org/10.1016/J.YEBEH.2020.106940”, which was not cited. The citation is ‘12’ and is in Section 1. Introduction, paragraph 3. There has been no change in the content of the paragraph; the only issue is that the reference is incorrect and does not reflect the true author (Yon et al.). Au Yang et al. can be removed, and reference 12 can be replaced with Yon et al.

Moreover, other medical comorbidities, including epilepsy [5], structural brain abnormalities [5], a history of brain surgery [5], febrile convulsion, status epilepticus and the use of combined (≥3) antiepileptic drugs (AEDs), have also been proposed as potential risk factors for PNES in patients with epilepsy [12].

The authors state that the scientific conclusions are unaffected. This correction was approved by the Academic Editor. The original publication has also been updated.
